# Tissue Remodeling in Chronic Eosinophilic Esophageal Inflammation: Parallels in Asthma and Therapeutic Perspectives

**DOI:** 10.3389/fmed.2017.00128

**Published:** 2017-08-07

**Authors:** Quan M. Nhu, Seema S. Aceves

**Affiliations:** ^1^Scripps Translational Science Institute, The Scripps Research Institute, La Jolla, CA, United States; ^2^Division of Gastroenterology and Hepatology, Department of Medicine, Scripps Clinic – Scripps Green Hospital, La Jolla, CA, United States; ^3^Division of Allergy and Immunology, Department of Medicine, Scripps Clinic—Scripps Green Hospital, La Jolla, CA, United States; ^4^Division of Allergy and Immunology, Department of Pediatrics, University of California, San Diego, La Jolla, CA, United States; ^5^Division of Allergy and Immunology, Department of Medicine, University of California, San Diego, La Jolla, CA, United States; ^6^Rady Children’s Hospital - San Diego, San Diego, CA, United States

**Keywords:** eosinophilic esophagitis, asthma, inflammation, tissue remodeling, fibrosis, structural cell dysfunction, corticosteroid, biologic therapy

## Abstract

Chronic eosinophilic inflammation is associated with tissue remodeling and fibrosis in a number of chronic T-helper 2 (Th2)-mediated diseases including eosinophilic esophagitis (EoE) and asthma. Chronic inflammation results in dysregulated tissue healing, leading to fibrosis and end organ dysfunction, manifesting clinically as irreversible airway obstruction in asthma and as esophageal rigidity, strictures, narrowing, dysmotility, dysphagia, and food impactions in EoE. Current therapies for EoE and asthma center on reducing inflammation-driven tissue remodeling and fibrosis with corticosteroids, coupled with symptomatic control and allergen avoidance. Additional control of Th2 inflammation can be achieved in select asthma patients with biologic therapies such as anti-IL-5 and anti-IL-13 antibodies, which have also been trialed in EoE. Recent molecular analysis suggests an emerging role for structural cell dysfunction, either inherited or acquired, in the pathogenesis and progression of EoE and asthma tissue remodeling. In addition, new data suggest that inflammation-independent end organ rigidity can alter structural cell function. Herein, we review emerging data and concepts for the pathogenesis of tissue remodeling and fibrosis primarily in EoE and relevant pathogenetic parallels in asthma, focusing additionally on emerging disease-specific therapies and the ability of these therapies to reduce tissue remodeling in subsets of patients.

## Introduction

Allergic inflammation has the capacity to recruit eosinophils to the site of inciting stimulus. Prolonged eosinophil infiltration can contribute to significant tissue injury, leading to maladaptive tissue remodeling and fibrosis. We will focus primarily on eosinophilic disorders associated with robust tissue remodeling, specifically eosinophilic esophagitis (EoE) and its relevant pathogenetic parallels in asthma.

## Clinical Features of Tissue Remodeling

The hypereosinophilic syndrome (HES)-associated tissue remodeling is arguably the most severe with cardiac damage leading to potential morbidity due to endomyocardial fibrosis. Asthma-associated airway remodeling occurs with epithelial denudation and goblet cell metaplasia, subepithelial fibrosis, angiogenesis, and smooth muscle hypertrophy (1). Remodeling is believed to be the mechanism to irreversible airway obstruction (2). EoE is an emerging chronic allergen-driven immune-mediated inflammatory disease that has been gaining recognition, with an increasing prevalence reaching 1 case per 1,000 persons (3–5). Chronic, unbridled inflammation in EoE leads to progressive esophageal fibrostenosis with rigidity and dysmotility with food impactions (6–9). Adult studies clearly demonstrate a natural history to stricture formation (6, 7). In both asthma and EoE, remodeling begins early in life, before the age of 6 years, and children with EoE can have histologic remodeling at as young as 2 years of age (2, 10).

Eosinophilic esophagitis is defined as a marked esophageal eosinophilic inflammation (≥15 eosinophils per high power field) that includes other inflammatory cells that likely contribute to remodeling such as mast cells, basophils, and adaptive as well as innate lymphoid cells (11–15). In the face of chronic antigen exposure and tissue damage, a progressive maladaptive esophageal tissue remodeling response causes clinical manifestations of dysphagia, food impactions, and, sometimes, spontaneous esophageal perforation (6, 16–20). In children, EoE often presents clinically as abdominal pain, nausea, vomiting, regurgitation, feeding difficulty, food aversion, weight loss, and failure to thrive; in adults, dysphagia and food impactions become more clinically prominent due to progression of esophageal dysfunction and fibrosis (13, 21). EoE severity has been associated with a lower body mass index, likely secondary to chronic nutritional deficit from recurrent dysphagia, food impaction, and food aversion (22). Although most EoE patients are well appearing, they often require a multimodal management approach that includes chronic medical treatment, dietary restriction, lifestyle changes, and repeated endoscopic diagnostic and therapeutic evaluations, creating a significant healthcare burden and impaired quality of life (18, 23–28).

In EoE, endoscopic features of remodeling vary between age groups. In children, features of esophageal pallor and furrows associate with histologic fibrosis and clinical dysphagia (29). In contrast, adult features of remodeling include concentric rings, narrowing, strictures, and the esophageal “pull” sign (30, 31). The narrowed and fibrostenotic esophagi are often the endoscopic features of adult EoE and can be intermittently observed in a subset of children (32). Functional readouts of esophageal rigidity include esophageal manometry and the novel application of the functional luminal imaging probe to assess esophageal rigidity and motility (33, 34). Indeed, esophageal rigidity predicts the risk of food impactions. Ultrasound studies in both adults and children show transmural esophageal thickening (35, 36). Similarly, CT scans of asthmatic airways demonstrate airway wall thickening even in children, while the HES heart can show increased cardiac muscle fibrosis with decreased chamber space (2).

### Histologic Features of Remodeling

Asthmatic airways demonstrate subepithelial fibrosis, with increased trichrome staining. The asthmatic epithelium demonstrates defective epithelial barrier function and loss of junctional proteins, with goblet cell metaplasia (2, 37). Airway epithelial barrier function is thought to regulate asthma pathogenesis (38). Subepithelial angiogenesis accounts for airway wall edema, while thickened airway smooth muscle causes airway hyperreactivity. On the basis of the findings in remodeled asthmatic airways, our lab sought to understand whether esophageal biopsies from children with severe EoE had histologic findings akin to the remodeled asthmatic airway. Indeed, histopathologic analysis has shown extensive cellular and extracellular remodeling changes in EoE (13, 21, 39, 40). Remodeling is manifested in the epithelium as basal cell hyperplasia, dilated intercellular spaces, and desquamation; and in the subepithelium as fibrosis, angiogenesis, and smooth muscle hyperplasia (16, 21, 41). The loss of barrier function is a cardinal feature of the EoE esophagus with decreased expression of desmoglein-1 and filaggrin in addition to decreased E-cadherin and claudin-1 (42–44).

## Molecular Mechanisms of Tissue Remodeling

### Interleukins and Cytokines Involved in Remodeling

Current concepts of tissue remodeling have centralized on cellular and extracellular matrix responses to repetitive tissue injury and ineffective tissue regeneration in the context of chronic inflammation (1, 13, 21, 45). It appears that the mechanisms of remodeling are similar in asthma and EoE (Table 1). IL-4 and IL-13 play pivotal roles in asthma pathogenesis (1, 46). Progress in EoE pathogenesis to date has focused mainly on IL-13 (42, 47). Allergen-mediated induction of IL-4, IL-5, and IL-13 promotes a T-helper 2 (Th2) immune response, resulting in eosinophil recruitment and activation. In addition, profibrotic factors such as TGFβ1 appear to play an important role in the remodeling associated with these allergic diatheses (21).

**Table 1:** Eosinophilic esophagitis and asthma: summary.

	Eosinophilic esophagitis	Asthma
Clinical manifestations of dysregulated tissue remodeling	Esophageal narrowing, strictures, rigidity, dysmotility, dysphagia, food impactions	Irreversible airway obstruction, dyspnea, wheezing, oxygen desaturations
Relevant pathogenic cytokines	IL-5, IL-13, TGFβ1	IL-4, IL-5, IL-13, TGFβ1
Relevant pathogenic chemokines	CCL26	CCL11, CCL24, and CCL26
Cellular manifestations	Epithelial desquamation, basal zone hyperplasia, subepithelial fibrosis, angiogenesis, smooth muscle cell hypertrophy	Epithelial denudation, goblet cell metaplasia, subepithelial fibrosis, angiogenesis, smooth muscle hypertrophy
Tissue mastocytosis	Yes	Yes
Structural cell alterations	Myofibroblast formation, smooth muscle cell hypertrophy, epithelial barrier dysfunction	Myofibroblast formation, smooth muscle cell hypertrophy, epithelial barrier dysfunction

IL-13 has emerged as a master regulator in EoE and drives the recruitment and activation of eosinophils *via* eotaxin-3/CCL26 and IL-5, further augmenting Th2 inflammation in the esophagus that can result in irreversible stricture formation (42, 47, 48). IL-13 contributes to the disruption of the epithelial barrier function, in part, *via* induction of calpain-14 that cleaves desmoglein-1 (49). Esophageal epithelial cells respond to IL-13 stimulation with STAT6-dependent expression of eotaxin-3/CCL26 that amplifies the chemotactic signals for further eosinophilic recruitment (47). IL-13 either alone or in combination with TGFβ1 can induce tissue fibroblasts to express periostin, further promoting eosinophil adhesion to fibronectin (50). IL-13 overexpression in an inducible transgenic murine model causes esophageal eosinophilia and stricture formation; turning off IL-13 overexpression to remove allergic inflammation reduces tissue eosinophilia but is unable to reverse the established esophageal stricture (48). In addition, GATA-1-null eosinophil-deficient IL-13 transgenic mice are able to develop esophageal tissue remodeling as evidenced by esophageal epithelial thickness, collagen deposition, and cellular hyperplasia (51). In contrast to IL-5, IL-13-mediated esophageal dysmotility and dysfunction *via* collagen deposition, angiogenesis, and epithelial hyperplasia can occur independently of eosinophilic inflammation (48, 51). In murine models of asthma, airway structural remodeling has been shown to persist even after complete resolution of allergic inflammation (52, 53).

IL-5 is a major cytokine that regulates eosinophilopoeisis and the trafficking, survival, and activation of eosinophils (54). Arguably, the best evidence for the role of IL-5 in human asthma is the success of humanized, monoclonal anti-IL-5 antibodies in treating eosinophilic asthma although their ability to decrease remodeling in human tissues is not clear. Peripheral blood from patients with active EoE have increased frequency of circulating activated eosinophils and IL-5-expressing CD4^+^ T cells, and peripheral blood mononuclear cells from EoE patients produce significantly more IL-5 compared to healthy controls when stimulated with house dust mites, ragweed, milk, *Aspergillus fumigatus*, or soy (55–59). Upregulated local expression of IL-5 promotes eosinophilic trafficking to the esophagus (60–62). Mice deficient in either IL-5 or eosinophils have diminished lamina propria collagen and fibronectin deposition in experimental EoE (62, 63). Esophageal strictures develop in IL-5-overexpressing transgenic mice, but not if these mice are also genetically deficient in eosinophils (48), demonstrating that the pro-remodeling effects of IL-5 are not intrinsic to this interleukin but, rather, through its capacity to recruit and activate inflammatory cells.

### Eosinophils and Other Immune Cells in Tissue Remodeling

Tissue inflammation in EoE is patchy and can be transmural, with immune cell infiltration and structural changes extending from the epithelium to the underlying muscle layers allowing multiple tissue layers to be directly exposed to the damage induced by inflammatory cells (39, 64–66). Epithelial barrier disruption activates a program of IL-33, TSLP, and eotaxin-3/CCL26 expression in EoE that promotes Th2 immune activation and eosinophil infiltration (12, 67–71). Eotaxin-3/CCL26 is a potent chemoattractant for eosinophils that is highly upregulated in esophageal biopsies and sera of EoE patients (72, 73); plasma levels of eotaxin-1/CCL11 and eotaxin-2/CCL24 are not increased in active EoE (73). In comparison, epithelial levels of CCL24 and CCL26, but not CCL11, are elevated in severe asthma (1, 74). Asthmatic eosinophils migrate better in response to *ex vivo* stimulation with CCL26 than CCL11 or CCL24 (75); in addition, CCL26 stimulation of asthmatic eosinophils demonstrates a biphasic migration pattern that potentially contributes to eosinophil-dependent pathogenesis of persistent asthma. IL-33 and TSLP can activate the recently discovered Th2-promoting group 2 innate lymphocytes (ILC2), which are enriched in active EoE and may promote remodeling *via* the expression of IL-5 and IL-13 (14). Infiltrating eosinophils further drive EoE inflammation *via* a multitude of mechanisms including degranulation, inflammatory, and profibrotic cytokine secretion such as IL-4, IL-5, IL-13, GM-CSF, and TGFβ1, and eosinophil extracellular trap formation, which correlates with inflammatory features such as white exudates in active EoE (40, 69). GM-CSF blockade reduces basal cell hyperplasia and epithelial remodeling in experimental EoE (76). Other eosinophil blocking strategies are also successful in EoE animal models including antibody blockade with anti-Siglec-F (63, 77). Although eosinophils infiltrate densely in EoE, their complex interactions with non-immune cells such as epithelial cells, fibroblasts, and smooth muscle cells and other immune cells such as mast cells, ILC2, basophils, T cells, and invariant natural killer T cells likely dictate the histologic and clinical remodeling outcomes of the disease (5, 13, 14, 40, 78).

Eosinophilic esophagitis and asthma are also characterized by tissue mastocytosis, which contributes to esophageal and airway dysfunction. Murine models of EoE, which are deficient in mast cells, show that mast cells contribute to smooth muscle cell mass (11). Mast cells are also reservoirs for profibrotic factors such as TGFβ1, and decreases in mucosal mast cell numbers are likely one mechanism by which fibrosis improves following therapy (79). Similarly, other tryptase-positive cells such as basophils have been implicated in EoE, and blocking the TSLP receptor diminishes basophil-induced complications such as food impactions in experimental EoE (12).

### Profibrotic Cytokines

Symptomatic EoE presents clinically as dysphagia, stemming from maladaptive esophageal tissue remodeling that results in fibrosis causing esophageal dysfunction and dysmotility. Eosinophils and mast cells are significant sources of TGFβ1, as previously identified in the esophagus of EoE patients and in the lungs of asthmatic patients (16, 79, 80). Eosinophils and eosinophil-derived products increase extracellular matrix production of fibronectin and collagen I in primary human esophageal fibroblasts and muscle cells in a process dependent on TGFβ1 and p38 signaling (81). TGFβ1 expression is elevated in the epithelium and subepithelium of adult and pediatric EoE patients (16, 82). TGFβ1 signaling induces collagen deposition and production of fibronectin and other extracellular matrix proteins; and blockade of the canonical TGFβ1 signaling pathway, Smad2/3, decreases remodeling in an oral ova murine EoE model (83). Also invoking the canonical TGFβ1 pathway, there is an increased epithelial and subepithelial expression of nuclear Smad2/3 in pediatric EoE patients. In addition, eosinophil-derived products, secreted products from eosinophil-fibroblast/muscle cell co-cultures, TGFβ1, or IL-13 altered esophageal muscle contraction in a feline EoE model (81). In a cohort of pediatric EoE patients, fibrosis was associated with eosinophilic degranulation in the epithelium as measured by staining for eosinophilic major basic protein, whereas fibrosis was not associated with the degree of esophageal eosinophilia, the number of mast cells, or mast cell degranulation (67). Kita and colleagues proposed that detection of eosinophil degranulation might be a more accurate assessment of EoE severity, based on their observations of marked deposition of eosinophil-derived neurotoxin in adult EoE biopsies (84).

In addition to its profibrotic effects, TGFβ1 can alter tissue contractility. TGFβ1 activates tissue fibroblasts, resulting in myofibroblast differentiation that further contributes to extracellular matrix deposition and collagen contraction (85). In addition, TGFβ1 induces primary esophageal smooth muscle cell contraction, a mechanism dependent on the canonical Smad2/3 pathway and phospholamban, a sarcoendoplasmic reticulum protein that regulates calcium flux, which is upregulated in EoE biopsies (79, 85). It is interesting to speculate if esophageal phospholamban plays a role akin to asthmatic orosomucoid like 3, which is clearly implicated in the pathogenesis of asthma.

TGFβ1 also has significant effects on the epithelium. It breaks down epithelial barriers in asthma by decreasing the expression of adhesion molecules. In EoE, remodeling has been associated with epithelial–mesenchymal transition, a TGFβ1-regulated process (86, 87). TGFβ1 significantly induces plasminogen activator inhibitor 1 (PAI-1)/serpinE1 in esophageal epithelial cells. Epithelial PAI-1 reflects the severity of histologic fibrosis and is also required for TGFβ1-induced expression of phospholamban and α-smooth muscle actin (αSMA) in esophageal fibroblasts, suggesting that it is part of the pathway to esophageal myofibroblast accumulation (88). Children with genotype TT at the TGFβ1 promoter have significantly elevated numbers of TGFβ1-positive cells, increased mast cells (but not eosinophils), more severe epithelial remodeling, and, when food sensitized, worse fibrosis than children of non-TT genotype (89).

Fibrosis may also occur independently of TGFβ1, as other profibrotic molecules such as CCL18 and fibroblast growth factor-9 (FGF9) are elevated in EoE tissue biopsies (90, 91) and not all adult subjects have elevated TGFβ1 (82, 91). CCL18 is similarly elevated in the bronchoalveolar lavage and sera of asthmatic patients and preferentially attracts Th2 cells and basophils (92). Eosinophil-derived major basic protein induces FGF9 production that can contribute to the fibroproliferative response in EoE (90). To our knowledge, the role of FGF9 in asthma has not yet been described.

### Mechanotransduction and Remodeling

There is accumulating evidence that mechanical signals (“mechanosignaling”) alter the function of structural cells in the airway and esophagus in a manner that can be independent of, dependent on, or synergistic with, inflammation (93–95). Our recently published data demonstrate that rigid matrix alters the gene expression profile of primary human esophageal smooth muscle cells toward a pathogenic profile similar to that induced by TGFβ1 (95). EoE fibroblasts from children and adults had increased αSMA and traction force when cultured on a rigid matrix (94). Airway epithelial cells respond to physical parameters such as compressive forces mimicking those seen in an edematous airway with increased production of disease relevant inflammatory markers such as endothelin and TGFβ2 and decreasing expression of barrier proteins (93). In addition, compression forces increase fibroblast expression of collagens. Asthmatic bronchial fibroblasts exhibit higher elastic modulus than control cells; TGFβ1-induced differentiation of bronchial fibroblasts into myofibroblasts is enhanced by increasing matrix stiffness (96, 97). Airway smooth muscle cell contraction induces the release of more active TGFβ1 (98). Methacholine-induced bronchoconstriction in the absence of inflammation is sufficient to induce airway remodeling in asthmatic patients (99). Taken together, these compelling data invoke a shift in the thought paradigm from focus almost exclusively on inflammation to one with an integrated focus on the mechanosignaling coupled to inflammation. Indeed normalization of mechanosignaling is likely required to effectively reduce the propagation of inflammation and dysregulated structural cell gene expression. Currently, it is not clear what direct or indirect effects there are on inflammatory cells cultured either in an environment that is rigid or compressed. However, it is well accepted that cells such as mast cells respond to physical insults such as scratching.

## Current and Emerging Therapeutics for Allergic Remodeling

Currently, there are no FDA-approved drugs indicated for the treatment of EoE. Some of the current treatment strategies and their effects on airway and esophageal remodeling are summarized below (5, 18, 28, 100, 101).

### Topical Corticosteroids

Topical esophageal corticosteroids constitute the most commonly utilized EoE therapy in children and adults. Similarly, inhaled corticosteroids are the most common agent used for persistent asthma. There has been relatively rapid accumulation of data for EoE since biopsies are procured regularly as part of disease monitoring. In contrast, airway biopsy is done in the context of clinical trials. Short-term studies in children have demonstrated that topical corticosteroids decrease fibrosis, VCAM-1, epithelial remodeling, subepithelial TGFβ1, and nuclear Smad2/3-positive cells in the subset of patients who have resolution of epithelial eosinophils following therapy (102). As such, it appears that in “responder” children, remodeling is in flux and can be reversed or improved with short-term therapy. Such treatment-responsive remodeling likely constitutes a physiologic rather than a pathologic process. In contrast, children who are “non-responders” to therapy, as defined by persistent esophageal eosinophilia despite therapy, have continued subepithelial fibrosis, vascular activation, and TGFβ1-expressing cells. Topical fluticasone treatment downregulates mRNA expression of eotaxin-3 and decreases the degree of eosinophilic and lymphocytic tissue infiltration in EoE esophagi (47, 103, 104). In addition, topical fluticasone treatment of EoE patients reduces IL-13 mRNA expression and reverses expression of 98% of IL-13-induced EoE transcriptome to the levels of healthy controls (47). EoE esophageal mucosal integrity is improved with topical fluticasone, as seen with normalization of expression of desmoglein-1 and filaggrin (105, 106). Peripheral blood eosinophils isolated from adult corticosteroid-treated EoE patients sustain their activated phenotype (107), but exhibit decreased CD18 surface expression, with resultant diminished adherence of eosinophils to ICAM-1, ICAM-2, and endothelial cells (108). Budesonide treatment of adult EoE patients results in a statistically significant reduction in absolute blood eosinophil count and serum levels of CCL17, CCL18, CCL26, eosinophil-cationic protein, and mast cell tryptase. In addition, the absolute blood eosinophil count changes correlate with esophageal eosinophil density (109, 110).

Since EoE is a chronic disease, chronic therapy seems warranted. Studying a group of 32 children over a mean of 5 years (maximum of 10 years) treated with corticosteroids, Rajan and colleagues showed that children with EoE who persistently respond well to therapy have significantly less fibrosis and lower endoscopic scores than children who respond suboptimally to therapy (10). The clinical reasons for differences in response to therapy are not clear. However, it is possible that a “remodeling first-inflammation second” EoE phenotype is less responsive to steroid therapy. It is also possible that mechanical alterations in the esophagus, such as rigidity, change the structural and/or inflammatory cell response to interventions. This concept is echoed in the adult literature where the fibrostenotic, dysmotile esophagus is substantially more resistant to topical therapy with corticosteroids (9, 111). In terms of endoscopic and symptoms severity, topical corticosteroids can improve the diameter of the strictured adult EoE esophagus and decrease the rate of food impactions (8, 112).

In asthma, the effects of inhaled corticosteroids on remodeling and the best remodeling endpoint to follow are not entirely clear (2). This is likely due to the paucity of repeated human airway tissue for study and the complexity of the pulmonary structure as branching occurs. In a murine model of allergen-induced asthma, corticosteroids prevent myofibroblast accumulation and peribronchial collagen deposition and fibrosis (113). In addition, corticosteroids can improve a subset of gene transcripts in asthmatic airway fibroblasts (2). Combination treatments with inhaled corticosteroids and long-acting β2-adrenergic receptor agonists together have demonstrated superior prevention of asthma exacerbations (114). Systemic corticosteroids used during severe asthmatic exacerbations exhibit variable responsiveness, thought related to the underlying asthma heterogeneity, for example, corticosteroid-responsive type 2-high airway inflammation-driven “concordant disease” versus corticosteroid-resistant type 2-low “discordant disease” (115–119). Although inhaled steroids can improve epithelial shedding, this is not a consistent finding. Studies of the reticular basement membrane thickening demonstrate improvements, but whether improvement in basement membrane thickening corresponds to improvements in asthma complications such as difficult-to-treat airway hyperreactivity or irreversible airflow obstruction is not clear. Although there is not a paucity of human tissue for study in EoE, the most clinically meaningful endpoint of remodeling is still unclear, although the best targets are likely to be fibrosis and early-onset esophageal rigidity.

Efficacy of topical corticosteroid therapy is dependent on mucosal drug delivery and esophageal mucosal contact time (120). Swallowed aerosolized corticosteroid has variable delivery, with oral viscous corticosteroid preparation achieving superior esophageal mucosal delivery and treatment efficacy (120). Emerging non-proprietary and proprietary formulations of corticosteroid are expected to improve treatment options, drug bioavailability, and treatment efficacy (120–124). While corticosteroid treatment for EoE is effective, there exists a significant number of EoE patients who do not respond to topical corticosteroid treatments (122, 125). It has been proposed that topical corticosteroids are unable to penetrate the deeper esophageal layers where significant eosinophilic inflammation and tissue remodeling and fibrosis are likely to take place. Oftentimes, biopsies are limited to the superficial layers and may offer an incomplete picture of the histologic response. Targeting the fibrotic tissue may offer enhanced corticosteroid uptake. Currently, a clinical trial for EoE is examining the effect of losartan, an angiotensin II receptor blocker used clinically for hypertension that also exerts anti-fibrotic effect through suppression of active TGFβ1 levels (126). Losartan has been shown to inhibit collagen I synthesis, resulting in improved distribution and efficacy of antitumoral agents (126). Another potential beneficial effect of anti-fibrotic therapy might involve an indirect improvement of structural cell dysfunction by reducing tissue rigidity. This is based on the novel observation by Aceves and colleagues that a rigid matrix induces morphologic and transcriptional changes in esophageal smooth muscle cells with increased collagen deposition and cellular hypertrophy (95). Similar subsequent work by Muir et al. demonstrated the role of matrix stiffness in modifying TGFβ1signaling and contractility of primary esophageal fibroblasts (94). Taken together, targeting inflammation-dependent and inflammation-independent, rigidity-dependent pathways may represent novel strategies to modulate tissue remodeling and fibrosis in EoE and beyond.

### Elimination Diets in EoE

In children, dietary modification to remove allergen-derived antigenic stimulation has been shown to reverse subepithelial fibrosis in EoE (127, 128). In addition, the combination of elimination diet and topical corticosteroids can decrease fibrosis in children (127). The effect of elimination diet on adult remodeling is not as clear.

### Biologic Therapy

Anti-IL-5 blockade with mepolizumab is safe and achieves significant reduction in circulating peripheral eosinophils and inflamed tissue eosinophilia (129–131). Even though IL-5 is a key regulatory cytokine of eosinophils that is upregulated in EoE, anti-IL-5 therapy using two different humanized monoclonal antibodies partially reduces tissue eosinophilia but does not alter esophageal fibrosis (132, 133). Although histologic or radiographic endpoints have not been systematically assessed in asthma, anti-IL-5 is effective in patients with severe, steroid refractory asthma and can be steroid sparing in patients with HES (134–136). In children with EoE, mepolizumab treatment decreases the numbers of tryptase-positive cells, IL-9-positive cells, and esophageal eosinophil–mast cell couplets (137).

Anti-IL-13 monoclonal antibody QAX576 significantly reduces esophageal eosinophilia and expression of EoE-related genes up to 6 months after treatment, but demonstrates only a trend for improved clinical symptoms (138). IL-13 blockade with a humanized monoclonal antibody RPC4046 significantly reduces esophageal eosinophilia and endoscopic features in EoE patients and also improves dysphagia; however, the effect is more prominent in steroid refractory EoE patients, suggesting that severe subjects may do well with anti-IL-13 therapy (139). This is consistent with the decrease in transcription of some remodeling genes including periostin for up to 6 months following treatment (138). Anti-IL-13 therapy also decreases markers of remodeling such as periostin and osteopontin in asthmatics, and subjects with higher serum periostin levels are more responsive to anti-IL-13 therapy (136, 140). Dupilumab, a blocker of both IL-4 and IL-13, may be of utility in asthma and EoE-associated remodeling (140–142).

## Conclusion

Both EoE and asthma are diseases that involve robust tissue remodeling as part of the disease processes with resultant end organ dysfunction in a subset of subjects. In asthma, the clinical complication is irreversible airway obstruction. In EoE, it is stricture formation. One mechanism to this complication is prolonged, unbridled inflammation that can occur due to lack of therapeutic intervention or the failure of therapies to adequately control disease progression. The presumed inflammatory signals are from infiltrating cells that respond to alterations in structural cell physiology such as decreased barrier function and the onset of chemokine production. However, other signals such as mechanical changes in the airways due to airway rigidity and epithelial contraction during repeated rounds of bronchoconstriction drive structural cells such as epithelium to generate inflammatory signals that could propagate inflammation and be unresponsive to standard anti-inflammatory therapies such as corticosteroids.

In addition to these issues, a number of additional considerations should be made when assessing the Th2-associated remodeling. The first issue is what parameters reflect remodeling most reliably? The second is the issue of pathogenic versus physiologic remodeling. The use of physiologic markers such as esophageal strictures or fixed airway obstruction likely represents an endgame of chronic disease and will likely be difficult to control. For this reason, one goal should be to find early markers of remodeling and control them. Of course, remodeling is also a normal process of wound healing that is necessary and required. What is not clear is how the shift from physiologic to pathogenic remodeling occurs. Possible explanations include disease duration, chronic inflammation, and/or mechanical signals such as tissue rigidity. Understanding the molecular mechanisms and the clinical phenotypes of these processes will be essential to better control allergic tissue remodeling and its consequences.

## Author Contributions

QN and SA reviewed the literature and wrote the manuscript.

## Conflict of Interest Statement

The authors declare that the research was conducted in the absence of any commercial or financial relationships that could be construed as a potential conflict of interest.
